# Orbital venous lymphatic malformations: case series on minimally invasive treatment approach

**DOI:** 10.22336/rjo.2025.45

**Published:** 2025

**Authors:** Ankita Ranjan1, Apjit Kaur Chhabra, Manoj Kumar

**Affiliations:** 1 Department of Ophthalmology, Sanjay Gandhi Post Graduate Institute of Medical Sciences, Lucknow, India; 2 Department of Ophthalmology, King George’s Medical University, Lucknow, India; 3 Department of Radiodiagnosis, King George’s Medical University, Lucknow, India

**Keywords:** orbital lymphangiomas, orbital venous lymphatic malformations, foam sclerotherapy, CON = Compressive Optic Neuropathy, USG = Ultrasonography, CT scan = Computed Tomography scan, MRI = Magnetic Resonance Imaging, mTOR = mammalian target of rapamycin, OK-432 = Picibanil

## Abstract

Orbital venous lymphatic malformations or lymphangiomas are choristomas of the orbit. Common in the pediatric population, the lesion is notorious for spreading into various anatomical spaces due to its infiltrative nature. Additionally, the vascular nature poses challenges to complete removal. Hemorrhages are not uncommon in the lesion, which may result in permanently compromised ocular function. Medical management involves injecting sclerosant into the cystic areas of the lesion, resulting in shrinkage and collapse, followed by total excision, which is the most suitable treatment option. The authors present a series of cases in which lymphangiomas have been completely excised using this minimal manipulative approach.

## Introduction

Orbital venous lymphatic malformations, previously known as lymphangiomas, are benign and localized malformations of the vascular and lymphatic systems. The lesion is common in the pediatric population, occurring in the head and neck region. Orbital venous lymphatic malformation constitutes about 0.3% to 4% of all orbital tumors and is estimated to have a prevalence of 1.1 to 5.3 per 10,000 live births, with equal incidences between males and females. These lesions are categorized as choristomas due to the absence of lymphatic channels in the orbit [1,2].

The common clinical presentation is proptosis, with or without dystopia, which depends on the lesion’s location in the orbit. Proptosis usually develops gradually and is painless, unless a hemorrhage occurs in the lesion, either spontaneously or following trivial trauma. Such patients present with rapid, painful, progressive proptosis, which needs urgent intervention due to the developing compartment syndrome and subsequent compressive optic neuropathy (CON) and corneal exposure. The slow-growing tumor may also result in impaired visual functions, including blepharoptosis, restriction of ocular motility, and compressive optic neuropathy [1-3].

The surgical treatment of lymphangiomas is challenging, due to the infiltrative, unencapsulated, and vascular nature of the tumor. Recurrences are common due to inadequate surgical excision. Another treatment modality is sclerotherapy, which has proven effective in treating macrocystic lesions (>2 cm) with lesser efficacy in microcystic lesions (<2 cm) [3]. Foam sclerotherapy is a relatively newer technique, and it is often the most favorable treatment option for lymphatic malformations of the head and neck region, which extend and occupy multiple anatomical spaces. Sclerotherapy can be performed either transcutaneously or transcatheterally, under fluoroscopic guidance [4,5]. Therefore, a multidisciplinary treatment approach is required for managing microcytic venous lymphatic malformations, which involves combining sclerotherapy with surgical debulking. The authors presented a case series of four patients with microcytic venous lymphatic malformation managed successfully with this minimally invasive treatment approach.

## Case 1

**Fig. 1:**
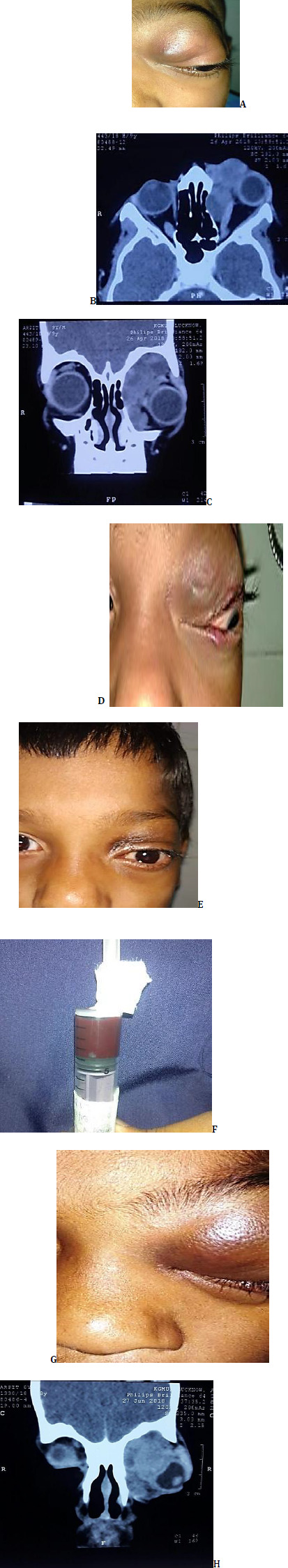
**A**. Clinical picture of the patient at presentation; **B, C**. Axial and coronal CT images illustrating an ill-defined, cystic lesion in the extraconal compartment of the left orbit, displacing the eyeball inferomedially; **D**. Clinical picture of the patient 4 weeks post-foam sclerotherapy; **E**. Clinical picture of the patient 2 weeks post-foam sclerotherapy, following trivial trauma; **F**. Axial CT image illustrating hemorrhage in the lesion (chocolate cyst); **G**. USG-Guided aspirate from the hemorrhagic cyst; **H**. Clinical picture of patient two months after surgical excision

A 9-year-old boy presented to the Oculoplasty clinic with a complaint of painless swelling over the left eyelid, which had gradually progressed over 7 years and was associated with proptosis and dystopia (**Fig. 1A**).

A bluish, ill-defined, non-tender, lobulated swelling, around 4.5x2.4 cm, was present in the superomedial aspect of the left orbit that had displaced the globe anteriorly and inferolaterally. Engorged veins were present on the surface with no signs of inflammation. On eyelid eversion, the palpebral and forniceal conjunctivae were erythematous with dilated and tortuous veins. The right orbit was found to be unremarkable.

Visual acuity was recorded as 20/63 in the right eye and 20/80 in the left eye, respectively. Fundus examination revealed a hyperemic optic disc with 360° blurred margins and an obliterated cup, suggestive of compressive optic neuropathy. Increased venous tortuosity was evident on the fundus. The posterior segment of the right eye appeared to be normal. The CT scan revealed a heterogeneously enhancing, infiltrative, lobulated lesion in the medial aspect of the left orbit, extending into the preseptal space of the eyelids (**Fig. 1B, C**).

Four weeks post a single round of sclerotherapy, there was a significant reduction in the size of the swelling, and the residual mass left was planned to be excised (**Fig. 1D**).

Unfortunately, two weeks post-sclerotherapy, the patient suffered blunt trauma, which led to haemorrhage into the lesion, worsening the dystopia, which was evident on CT (**Fig. 1E, F**). Surgery was deferred, and USG-guided aspiration of a blood-filled cyst was done (**Fig. 1G**). After two months, the lesion was surgically excised through a vertical lid split approach. The patient responded satisfactorily to the management and, in subsequent follow-up visits, showed no signs of recurrence (**Fig. 1H**).

## Case 2

**Fig. 2:**
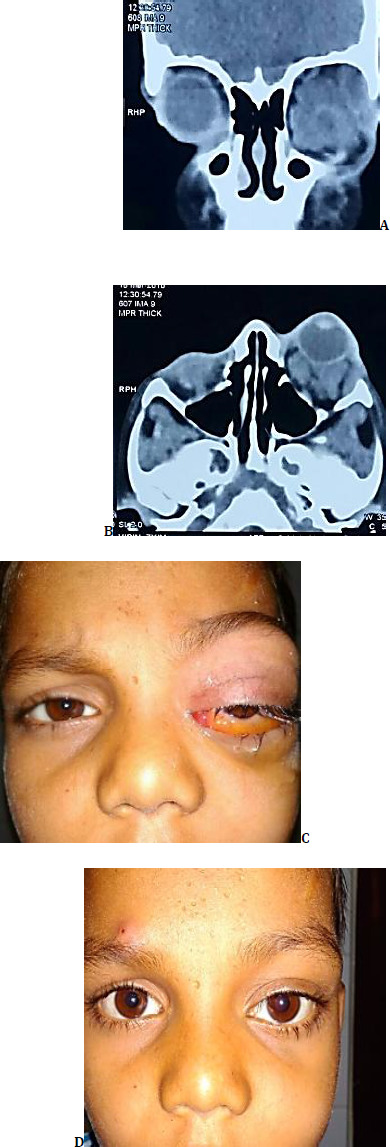
**A**. Clinical picture of the patient at presentation; **B, C**. CT image illustrating an ill-defined, cystic lesion in both intra- and extra-conal space of the left orbit pushing the globe anteriorly and slightly inferiorly; **D**. Clinical picture of the patient three months after treatment

A 7-year-old boy, who presented to the Oculoplasty clinic with slowly developing swelling in the left periorbital area associated with forward protrusion of the globe (**Fig. 2A**).

There was axial proptosis of the left eye, accompanied by lagophthalmos. The left globe was frozen, and the overlying skin of the eyelids had prominent and engorged veins along with erythema and bluish discoloration. Conjunctiva was chemosed and hyperemic with signs of exposure keratopathy. Relative afferent pupillary defect was elicited in the left eye. The right orbit was found to be unremarkable. Visual acuity in the right eye was 20/20, and in the left eye was reduced to 20/100. Dilated fundus examination showed signs suggestive of optic neuropathy in the left eye, whereas the right eye was normal. The CT image was suggestive of lymphangioma, which had both intra- and extra-conal components, pushing the left globe anteriorly and slightly inferiorly (**Fig. 2B, C**). Some part of the lesion had made its way infraorbital into the subcutaneous area of the left cheek. Hence, consultation with the faciomaxillary surgeon was done simultaneously.

To begin with, a multidisciplinary approach was employed, involving temporary tarsorrhaphy of the left eye and ultrasonography-guided sclerotherapy of both the orbital and cheek lesions. Two weeks post-sclerotherapy, when the size of the lesion was substantially reduced, tarsorrhaphy sutures were removed. The residual orbital component was carefully resected via a conjunctival approach. The patient was then referred to the faciomaxillary surgeon for the management of the infraorbital component. By the end of three months, the visual alignment was nearly restored, visual acuity had improved to 20/40, and ocular movements were free and complete in all directions of gaze (**Fig. 2D**).

## Case 3

**Fig. 3:**
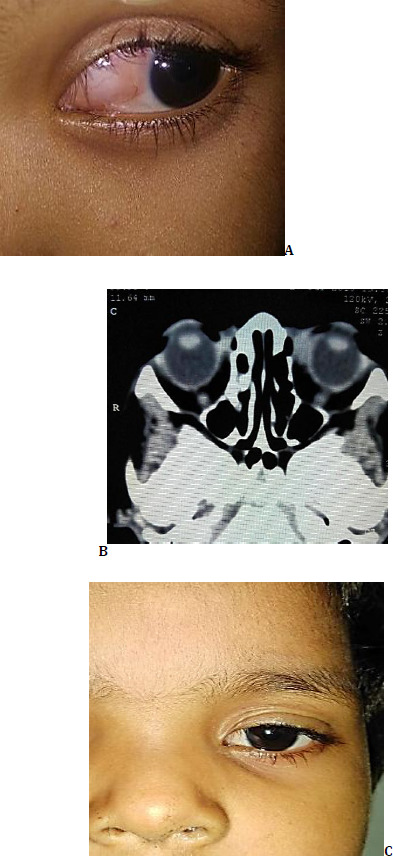
**A**. Clinical picture of the patient at presentation; **B**. CT Imaging illustrating an ill-defined, heterogenous lesion, in the medial side of the left orbit, extending into the intraconal space; **C**. Post-op clinical picture of patient at one-month follow-up

A 4-year-old girl, who was referred to the Oculoplasty clinic from an urban health care center with the tentative diagnosis of left medial orbital dermoid. The patient’s attendant offered the history of an episode of bleeding following some trivial trauma about a month back, which resulted in an increased size of the swelling (**Fig. 3A**).

A medial conjunctival lesion with an undefined posterior margin was found. The swelling had a bluish hue, with dilated telangiectatic blood vessels visible over the surface and extending into the substance. There was no malalignment of the visual axis or dystopia. Visual acuity was 20/20 in both eyes, and dilated fundus examination was unremarkable. Left eye ocular movements were slightly restricted in adduction. CT imaging revealed an ill-defined, heterogeneous, non-contrast-enhancing lesion in the medial side of the left orbit, extending beyond the equator and encompassing the medial rectus muscle, suggestive of lymphangioma (**Fig. 3B**).

The patient underwent a single round of CT-guided foam sclerotherapy in multiple macrocystic spaces. Six weeks postoperatively, the lesion had partially resolved, and extraocular movements were fully restored. Surgical excision through a conjunctival approach was performed. The patient was followed for over 12 months and showed no signs of recurrence or any episodes of bleeding thereafter (**Fig. 3C**).

## Case 4

**Fig. 4:**
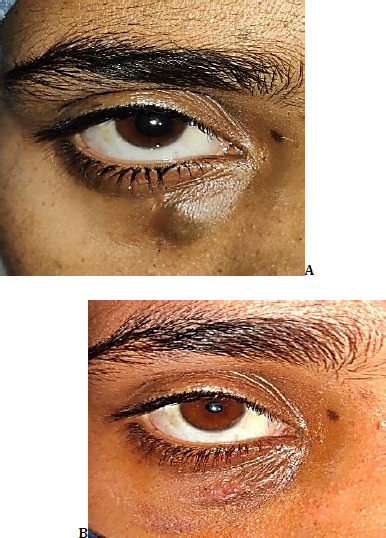
**A**. Clinical picture of the patient at presentation; **B**. Clinical picture of the patient six weeks after sclerotherapy, followed by total surgical excision

A 19-year-old female presented to the clinic with a swelling inferior and medial to the right lower lid, which had gradually progressed over 6 months (**Fig. 4A**).

The patient had previously been treated conservatively as chronic dacryocystitis in a rural health care center for the same, and was referred to the Oculoplasty clinic when no signs of improvement were noted.

The swelling was cystic, non-tender, and ill-defined with a bluish discoloration of the overlying skin. Regurgitation on pressure was negative. Syringing was performed, following the previous diagnosis, to rule out any obstruction at the level of the nasolacrimal duct or canaliculi, but it revealed a patent drainage tract. Any association with the lacrimal drainage system was thus ruled out, and secondary differentials were considered.

The visual acuity was 20/20 in either eye, and the anterior segment examined was unremarkable. There was neither complaint of watering nor any discharge from the eye of the affected side. There was no complaint of pain in the affected area. CT revealed a heterogeneous, ill-defined, infiltrative, and cystic lesion in the inferomedial part of the right orbit, located in the extraconal compartment, consistent with a lymphangioma.

The patient was then planned for transcutaneous ultrasound-guided sclerotherapy, which went uneventfully. The lesion substantially regressed over two weeks and was then completely resected thereafter. By the end of six weeks, no residual tumor could be detected, and to date, it has shown no signs of recurrence (**Fig. 4B**).

## Discussion

Lymphangiomas are locally aggressive, nonencapsulated, multicystic tumors that grow insidiously. The infiltrative nature often results in a breach of anatomical boundaries. These consist of endothelial-lined channels, which are not true lymphatic vessels; therefore, the term “venous lymphatic malformation” is more accurate. The International Orbital Society has grouped the vascular malformations based on hemodynamic properties into a) Type 1 or no-flow malformations, b) Type 2 or venous flow malformations, and c) Type 3 or arterial malformations. Lymphangiomas are hemodynamically isolated, with minimal blood flow velocity, and have been grouped under no-flow malformations. They are neither pulsatile nor associated with a bruit, and their size is independent of postural head changes. Based on their predominant components, they are also classified as either venous-dominant or lymphatic-dominant. These are also categorized based on their cyst size as follows: a) microcystic (<2 cm), b) macrocystic (>2 cm), previously known as cystic hygromas, or mixed type [1-4].

Orbital lymphatic malformations generally manifest in early childhood, 43% before the age of 6 years and 60% before the age of 16. In children, visual monitoring is of utmost importance, as significant ptosis can lead to vision deprivation and amblyopia. At the same time, compressive optic neuropathy can cause permanent visual loss, necessitating prompt treatment of the underlying pathology [6,7].

The diagnosis is made through a thorough clinical examination, supplemented by radiological investigations. Differential diagnosis includes dermoid, hemangioma, rhabdomyosarcoma, or lymphoma. Ultrasonography (USG) is a sensitive yet straightforward test that can be performed as an initial investigation. Lymphangiomas on ultrasound (USG) appear as high-amplitude, widely separated echoes, suggestive of large fluid-filled spaces. However, the margins of the lesions cannot be delineated on USG. Computed tomography scans (CT scans) have the upper hand as the imaging modality due to their capability to correlate well anatomically. On CT scan, lymphangiomas appear as poorly defined, infiltrative, multicystic lesions that are hyper-attenuating, with slight or no contrast enhancement. Cysts with hemorrhage show rim enhancement. These imaging characteristics were consistent with our cases, which guided our preoperative assessment and eased the planning of the management. Magnetic Resonance Imaging (MRI) is now considered superior to other modalities in terms of contrast resolution and spatial localization. The ability to identify feeder vessels without the need for contrast is exclusive and helps to rule out lymphangioma. T1-weighted images with fat suppression are best suited to detect blood-filled cystic spaces that appear hyperintense [7,8].

Contrary to the ease of their diagnosis, the management of the orbital venous lymphatic malformation is devious, the reason being their diffuse infiltrative nature and propensity to develop spontaneous hemorrhage. These malformations never involute. However, observation alone can be considered in pediatric patients below 36 months of age to avoid exposing them to the risks of general anesthesia. Surgery alone comes with challenges due to the infiltrative and vascular nature of the lesion. Maintenance of esthetics and proximity to vital structures also determine the surgical plan. Unfortunately, partial excisions are not uncommon, and frequent recurrences necessitate repeated surgery [9,10].

To overcome this limitation, a wide range of adjunct non-procedure treatments have been tried, including subjecting the tumor to intralesional sclerosant, either with corticosteroids, PDE-5 inhibitors such as systemic sildenafil, or mTOR inhibitors like sirolimus, as well as carbon dioxide laser ablation of the cysts and pre-operative embolization. Of these, sclerotherapy is widely accepted and safe in patients with orbital venous lymphatic malformations. Some agents used for sclerotherapy include OK-432 (Picibanil), sodium tetradecyl sulfate, doxycycline, ethanol, pingyangmycin, and bleomycin [11,12]. Usually performed under ultrasound guidance, this procedure involves injecting the sclerosant agent into the cystic spaces of the lesion. This results in sterile inflammation of the vessel wall and subsequent collapse of the cysts and their extensions. The size of the lesion reduces, and it can be excised surgically with little exploration. Several studies have shown that cystic lesions respond effectively to sclerotherapy, with even a single dose often sufficient for complete resolution. However, it has also been noted that macrocystic lesions respond better to sclerotherapy than the microcystic ones; hence, the rationale of combining sclerotherapy with surgical excision forms the background of “Minimally Invasive Treatment Approach” to macrocystic type of venous lymphatic malformations, which has been put forward through this case series [1,3-15].

In the first case, the patient presented with blepharoptosis and proptosis, which have been reported as the most common presenting symptoms in past studies. The second patient also presented with proptosis with associated lagophthalmos and facial extension of the lesion. Both patients had developed signs of compressive optic neuropathy, which warranted early treatment. USG-guided foam sclerotherapy using sodium tetradecyl sulphate combined with excision of the lesion proved successful. The third case was instead a superficial lesion, but taking into consideration the history of past episodes of bleeding, a minimally invasive treatment modality was planned. The fourth case was again a superficial lymphangioma without any deeper extension, which responded well to sclerotherapy and surgical excision subsequently.

## Conclusion

In this case series, we concluded that a minimally invasive approach, combining sclerotherapy with surgical excision, is a practical treatment option for venous lymphatic malformations.

Although MRI is the diagnostic modality of choice, CT itself is necessary and can detect the vascular component of the tumor easily and serve as a guide for the surgeons for safe resection.

Randomized controlled trials are required to conclude the efficacy of the minimal approach to treatment.

## References

[1] Woo YJ, Kim CY, Sgrignoli B, Yoon JS (2017). Orbital Lymphangioma: Characteristics and Treatment Outcomes of 12 Cases. Korean J Ophthalmol.

[2] Guinto G, Guinto-Nishimura Y (2014). Orbital lymphangiomas. World Neurosurg.

[3] Russin JJ, Rangel-Castilla L, Kalani MY, Spetzler RF (2015). Surgical management, outcomes, and recurrence rate of orbital lymphangiomas. J Clin Neurosci.

[4] Lee KH, Han SH, Yoon JS (2015). Successful treatment of orbital lymphangioma with intralesional bleomycin and application of continuous negative pressure. Korean J Ophthalmol.

[5] Nassiri N, Rootman J, Rootman DB, Goldberg RA (2015). Orbital lymphaticovenous malformations: Current and future treatments. Surv Ophthalmol.

[6] Bagrodia N, Defnet AM, Kandel JJ (2015). Management of lymphatic malformations in children. Curr Opin Pediatr.

[7] Bilaniuk LT (2005). Vascular lesions of the orbit in children. Neuroimaging Clin N Am.

[8] Khunger N (2010). Lymphatic malformations: current status. J Cutan Aesthet Surg.

[9] Garcia DD, Heran MK, Amadi AJ, Rootman J (2011). Low outflow distensible venous malformations of the anterior orbit: presentation, hemodynamic factors, and management. Ophthalmic Plast Reconstr Surg.

[10] Zhou Q, Zheng JW, Mai HM, Luo QF, Fan XD, Su LX, Wang YA, Qin ZP (2011). Treatment guidelines of lymphatic malformations of the head and neck. Oral Oncol.

[11] Adams DM, Trenor CC, Hammill AM, Vinks AA, Patel MN, Chaudry G, Wentzel MS, Mobberley-Schuman PS, Campbell LM, Brookbank C, Gupta A, Chute C, Eile J, McKenna J, Merrow AC, Fei L, Hornung L, Seid M, Dasgupta AR, Dickie BH, Elluru RG, Lucky AW, Weiss B, Azizkhan RG (2016). Efficacy and Safety of Sirolimus in the Treatment of Complicated Vascular Anomalies. Pediatrics.

[12] Poonyathalang A, Preechawat P, Jiarakongmun P, Pongpech S (2008). Sclerosing therapy for orbital lymphangioma using sodium tetradecyl sulfate. Jpn J Ophthalmol.

[13] Hill RH, Shiels WE 2nd, Foster JA, Czyz CN, Stacey A, Everman KR, Cahill KV (2012). Percutaneous drainage and ablation as first line therapy for macrocystic and microcystic orbital lymphatic malformations. Ophthalmic Plast Reconstr Surg.

[14] Gandhi NG, Lin LK, O’Hara M (2013). Sildenafil for pediatric orbital lymphangioma. JAMA Ophthalmol.

[15] Parentin F, Borzaghini L, Perissutti P (2001). The role of ultrasonography in the diagnosis of orbital lymphangiomas. Ophthalmologica.

